# An optimized postsurgery follow-up strategy for patients with esophageal cancer: a cohort study

**DOI:** 10.1097/JS9.0000000000000827

**Published:** 2023-11-02

**Authors:** Zihang Mai, Jiaxin Xie, Changsen Leng, Xiuying Xie, Jing Wen, Hong Yang, Qianwen Liu, Jianhua Fu

**Affiliations:** a Department of Thoracic Surgery, Sun Yat-sen University Cancer Center; b State Key Laboratory of Oncology in South China, Collaborative Innovation Center for Cancer Medicine; c Guangdong Esophageal Cancer Institute, Guangzhou; d National Cancer Center, Chinese Academy of Medical Sciences and Peking Union Medical College, Beijing, People’s Republic of China

**Keywords:** esophageal cancer, postsurgery follow-up, recurrence

## Abstract

**Background:**

After radical surgery, patients with esophageal cancer should undergo long-term surveillance of disease relapse. However, the optimal follow-up strategy remains to be explored.

**Method:**

A total of 4688 patients were recruited. Recursive partition analysis was applied to develop recurrence risk stratification for patients. The follow-up strategies of each stratification were developed based on monthly recurrence probability and validated by bootstrap validation and an external dataset. A Markov decision-analytic model was constructed to evaluate the cost-effectiveness of the follow-up strategies.

**Results:**

Patients were stratified into four groups according to four pathological features. The authors applied a random survival forest to calculate the monthly recurrence probability of each group. Based on the temporal distribution of recurrences, the authors further established surveillance strategies for four groups. The strategies were validated as optimal protocols by bootstrap resampling and another dataset. Markov cost-effective analysis indicated that our recommended strategies outperformed the mainstream protocols from guidelines. Using less than 12 visits across the first 5 years on average, our follow-up strategies were more efficient than the NCCN recommended strategies (14 visits average). Our results also supported the computerized tomography from the neck to the upper abdomen as a routine examination and PETCT of distant metastasis for some groups with high risks.

**Conclusion:**

Our study provided data-driven evidence of personalized and economic follow-up strategies for esophageal cancer patients and shed light on follow-up optimization for other cancer types.

## Introduction

Highlights

- We develop and validate a novel postsurgery recurrence risk stratification for patients with esophageal cancer based on large real-world cohorts.
- A recurrence-risk-based follow-up strategy is designed for esophageal cancer patients with different recurrence risks, using 17% fewer follow-up visits to achieve efficient and economical recurrence surveillance than the NCCN guidelines recommend.
- In addition to the computational tomography of the thorax and upper abdomen as recommended by the NCCN guidelines, our data suggests the computational tomography of the neck should be a routine examination for recurrence surveillance and PETCT for patients at the N3 stage.

Esophageal cancer (EC), the sixth leading cause of cancer-related death, is a worldwide health threat^1^. For resectable EC, the standard treatment is esophagectomy, and neoadjuvant therapies are optional for local advanced diseases^2^. Despite receiving radical treatment, nearly a half of patients undergo postsurgery recurrence, threatening patients’ lives again^3–5^. Therefore, post-treatment surveillance is critical for early detection of disease recurrence as minimal and restricted lesions may be more sensitive to salvage treatments^6,7^. On the other hand, the intervals of recurrence surveillance might affect the observation of endpoints used in clinical trials, especially the progress-free survival^8^. Therefore, effective follow-up schedules might contribute to the development of clinical research on EC patients.

Current follow-up guidelines, mostly on the ground of expert experiences gained from clinical trials, recommended that EC patients should receive lifelong surveillance, including repetitive physical and radiological examinations averagely per 3–6 months^2,9,10^ in the first 2 years and per 6–12 months hereafter. However, most of them were empirical, and few data supported the effectiveness of these guidelines^2^. Moreover, the risk of EC recurrence might be time-varying^10–12^, so identical follow-up intervals as the guidelines recommended might be suboptimal. The characterization of the temporal dynamics of EC recurrence is pivotal for tailoring follow-up intervals, but previous data from clinical trials may be incomplete due to the limited sample sizes and strict inclusions^10–12^. In the context of lacking prospective trials, investigations based on real-world cohorts are needed. Furthermore, current follow-up guidelines are unaware of the distinct recurrence risks among patients with different clinicopathological backgrounds^13–17^. Those one-size-fits-all strategies recommended by guidelines seem to be burdensome for some patients. How to stratify patients by different recurrence risks remains an open question in customizing follow-up strategies^18^.

To develop a recurrence-risk-based follow-up strategy for EC patients, we identified well-characterized pathological features to construct risk stratifications for patients. Next, we estimated the monthly probability of tumor recurrence for each patient groups and established effective and economical follow-up arrangements for different groups. Our study also provided a flexible analysis paradigm for prognosis stratification and individualized surveillance on other cancer types.

## Methods

### Patient selection

This discovery set of this study was comprised of 3878 histologically proven, EC patients diagnosed between 2008 and 2018 in our high-volume cancer center. Selection criteria were listed as below:

#### The inclusion criteria contained:

(1) Patients aged between 18 and 80 years old; (2) Pathological diagnosis as esophageal squamous cell carcinoma or adenocarcinoma; (3) Patients underwent esophagectomy and received R_0_ resection.

#### Exclusion criteria contained:

(1) Patients with secondary primary tumor; (2) Patients who died within 30 days after surgery or died of postoperation complications; (3) Patients missing essential clinical information, such as the operation record, pathological diagnosis, and follow-up data; (4) Patients with radiologically or histologically confirmed distant metastasis (DM); (5) Patients previously underwent endoscopic mucosal resection or definitive radiochemotherapy as initial treatment.

An independent cohort (*N*=810, diagnosed between 2007 and 2014) was selected for validation under the same criteria from the database of another institution. The work has been reported in line with the strengthening the reporting of cohort, cross-sectional and case–control studies in surgery (STROCSS) criteria^19^.

### Treatment and follow-up

Before treatment, all patients underwent detailed evaluations and were staged by experienced thoracic oncologists following the 8th American Joint Committee on Cancer (AJCC) staging manual^20^. Esophagectomy and two-field lymphadenectomy were performed by experienced surgeons. After surgery, patients were recommended for routine follow-up with an interval of 3–6 months in the first 2 years and every 6–12 months hereafter. Additional outpatient visits were performed on patients’ complaints of recurrence-associated symptoms^2^. More details of the follow-up are described in the Supplementary Note (Supplemental Digital Content, link: http://links.lww.com/JS9/B257).

### Data collection and definition of clinicopathological variables

The clinical data was collected from our EC-specific database using the established criteria. Briefly, our big-data research system automatically recognized medical reports and transformed them into structured data. Two researchers manually collated the data by reviewing the medical reports. In total, 51 clinicopathological variables were included (Table S1, Supplemental Digital Content, link, http://links.lww.com/JS9/B257). Among those variables, lymphvascular invasion (LVI) was defined as the presence of tumor cells within an endothelium-lined space^13^. Perineural invasion (PNI) was described as tumor cells within every layer of the peripheral nerve sheath^14^. The lymph node ratio (LNR) was proposed as the ratio of the number of metastatic lymph nodes to the total number of examined lymph nodes^17^. Localregional relapse (LRR) was tumor recurrences at anastomotic stroma and regional lymph nodes defined by the AJCC manual^20^, while DM recurrences at other organs and nonregional lymph nodes.

Follow-up data was collected via outpatient visits, online consultations, or telephone calls, survival endpoints were defined according to the commonly used criteria^21^.

### Construction of recurrence-risk stratification and follow-up strategies

We devised a two-step analytic approach to define patient groups with the largest prognostic differences in the discovery set (Figure S2A). Setting DFS as the primary endpoint, we conducted recursive partitioning analysis based on clinicopathological parameters mentioned above (Table S1, Supplemental Digital Content, link: http://links.lww.com/JS9/B257) to grow a decision tree that partitioned patients into subpopulations with the most distinct DFS. To reduce the redundancy of the model, a logrank test was performed to iteratively compare the survival differences of each group pair. The two groups with similar prognosis were combined, and the combined group served as a candidate of the next round of the logrank test. This procedure stopped until all the remaining groups were different enough in DFS (*P* value <0.001 and *P*_adjust_ <0.01). The rules were verified using bootstrap validation with 70% downsampling in the discovery set. The validation set was also used to confirm the prognosis differences of the self-defined groups.

The random survival forest (RSF) model was used to estimate time-specific recurrence probabilities for each group and arrange the follow-up schedule according to accumulated recurrence risk. The computational methods were detailed in the Supplementary Note (Supplemental Digital Content, link, http://links.lww.com/JS9/B257).

### Comparison of different follow-up strategies

The effectiveness of surveillance strategies was measured by the delayed detection time (DDT), which was defined as the duration from the recurrence occurrence to the nearest coming follow-up. For instance, if a patient was diagnosed as recurrence in the 12th month but the next planned visit was in the 14th month, the DDT for this patient was 2 months.

We also compared our strategy with the mainstream follow-up strategies proposed by National Comprehensive Cancer Network (NCCN) guidelines^22^ and some phase III randomized clinical trials^9,23–27^:

1. The surveillance protocol used in the CROSS trial^24^: every 3 months in year 1; every 6 months in year 2; once per year in year 3–5 (nine visits in total, CROSS strategy for brief).
2. The surveillance protocol in the NEOCRT5010 and OEO2 trials^23,26^: every 3 months in year 1; every 6 months in year 2–5 (12 visits in total, 5010 strategy for brief).
3. The most intensive surveillance is recommended by the NCCN: every 3 month in the first 2 years; every 6 months in year 3–5 (14 visits in total, NCCN strategy for brief).
4. The surveillance protocol used in NCT03001596^27^: the first visit at the first month, then every 3 months in year 1–2; every 6 months in year 3–5. (14 visits in total, NCT1596 for brief).Furthermore, the Markov decision-analytic model was constructed to simulate the life cycle of patients undergoing recurrences and compare the cost-effectiveness of the follow-up strategies (Figure S5).

### Statistical analysis

All patients were followed up for mortality until 31 August 2021 and *P* values for survival analysis were calculated by the logrank test. The median follow-up time was estimated by the reverse Kaplan–Meier method^28^. The Wilcoxon rank sum test was used to compare two groups of continuous variables. Fisher’s exact test was used to test for association between categorical variables. Two-sided *P* values were considered to be significant below 0.05, unless specified. All analyses were completed in R (version 4.0.2).

## Results

### Overview of the cohort

In total, 3878 patients in the discovery cohort and 810 patients in the validation cohort were included. The baseline clinicopathological characteristics of patients in two datasets were summarized in Table S2. In the discovery set, 78.9% of patients were male, the median age at diagnosis was around 60 and 18.3% received neoadjuvant therapy before surgery, similar to previous reports^29^. 18.8% of patients received oncologist-recommended adjuvant therapy. After a median follow-up duration of 57.5 months (95% CI: 54.7–59.8), there were 39 283 visits (median: 11 IQR: 7–13). Retrieving the medical records and imaging reports, we comprehensively analyzed 19 358 CT of the neck, chest, and upper abdomen, 4526 ultrasonography of the neck, 1219 MRI of the chest, 1897 positron emission tomography CT (PETCT), 4810 endoscopy or endoscopic ultrasonography and 10 558 pathological examination reports, including pretreatment biopsy, pathological reports of surgical specimen, and surveillance biopsy.

**Figure 1:**
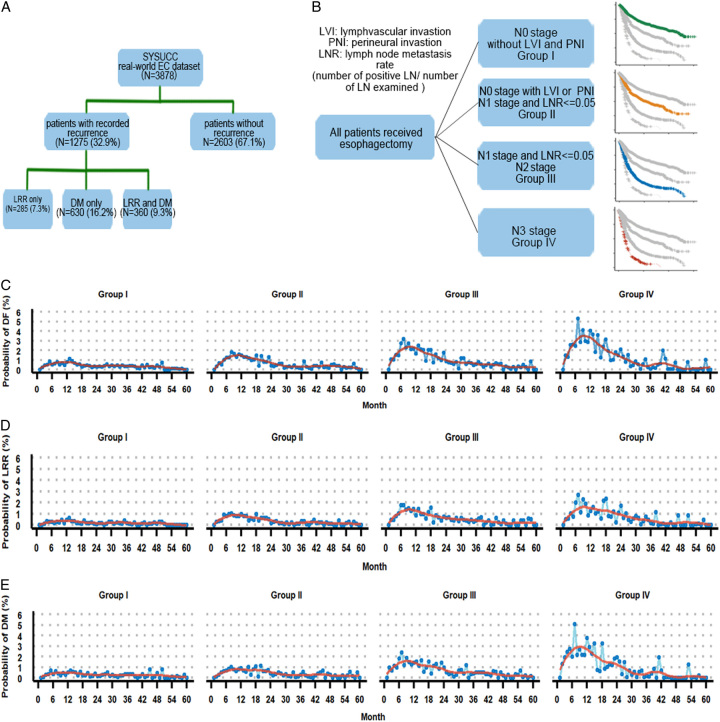
Overview and temporal distribution of recurrence probabilities in different groups. (A) Overview of recurrence patterns of our real-world EC datasets. (B–D) Temporal distribution of recurrence probabilities after surgery. Disease failure (B); local and regional recurrence (C); distant metastasis (D).

Supported by radiological and histological evidence, we observed 1275 patients undergoing disease recurrences, among them 285 had LRR, 630 had DM and both LRR and DM occurred in 360 patients (Fig. 1A). For patients with disease relapses, the median time to first LRR and DM was 12.8 months and 14.7 months, respectively.

### Recurrence probabilities of different prognosis related groups

Recurrences of EC did not occur at fixed probabilities, and the temporal features remained to be explored^10,30^. To address this issue, we used the RSF model to calculate the monthly recurrence probability of patients. We first asked whether there existed temporal differences in disease recurrences in patients receiving neoadjuvant therapy or not. As shown in Figure S1A (Supplemental Digital Content, link: http://links.lww.com/JS9/B257), the RSF-based recurrence-risk curves exhibited a remarkable overlap. Sensitivity analysis using another method named spline regression showed a similar trend of two treatment modalities (Figure S1B), Supplemental Digital Content, link: http://links.lww.com/JS9/B257, indicating neoadjuvant therapy reduced overall relapse risk but not delayed the tumor recurrence^3^.

As displayed in Figure 1B, we further used pathological features to stratify patients into different groups with recurrence risks regardless of their neoadjuvant therapy statuses: group I (T_any_N_0_, without LVI and PNI); group II (T_any_N_0_, with LVI or PNI; T_any_N_1_, with LNR≤0.05); group III (T_any_N_1_, with LNR>0.05; T_any_N_2_), and group IV (T_any_N_3_). As shown in Figure S1C (Supplemental Digital Content, link, http://links.lww.com/JS9/B257), 5-year recurrence probabilities for groups I to IV were 23.9, 40.9, 61.9, and 76.3%, respectively. The parameters reflecting basic body functions were comparable across the four groups (Table S3). Consistent with the trend of recurrence probability, the site-specific metastasis rate increased from group I to IV, with the exception that the lung metastasis rate of group III ranked the highest across the four groups. Additionally, the RSF-estimated survival curves showed a large overlap with the real-world observation calculated by the Kaplan–Meier method, indicating that the RSF approach could precisely describe the real recurrence risk (Figure S1D, Supplemental Digital Content, link: http://links.lww.com/JS9/B257).

We further verified the differences in the recurrence risk of the four groups. Among 1000 trials of bootstrap sampling, 99.2% of simulations in the four groups show significant differences in recurrence risks with each other, indicating the robustness of the groups (Figure S2D). Moreover, we compared the four groups in the validation cohort and confirmed their distinct prognoses (Figure S2C).

Next, monthly recurrence probabilities were estimated for each groups using RSF. For patients in group I, the relapse incidence was low and the fitted curves for all endpoints were smooth without remarkable peaks (Fig. 1C–E). As for patients with more advanced diseases (groups II–IV), the recurrence risk increased steeply towards a peak at about the 7th month after surgery, and the curves declined down to a half of the peak at about the 24th month (Fig. 1C).

As for different patterns of recurrences, the peak time of LRR occurred at around 7th month and was maintained at a high-risk plateau until 18th month after surgery, which was consistent across groups II–IV. We observed a similar phenomenon at the curves of DM and an additional miniature peak of DM in group IV at about 42th month, not of LRR (Fig. 1E).

### Construction of follow-up strategies for different groups

**Figure 2:**
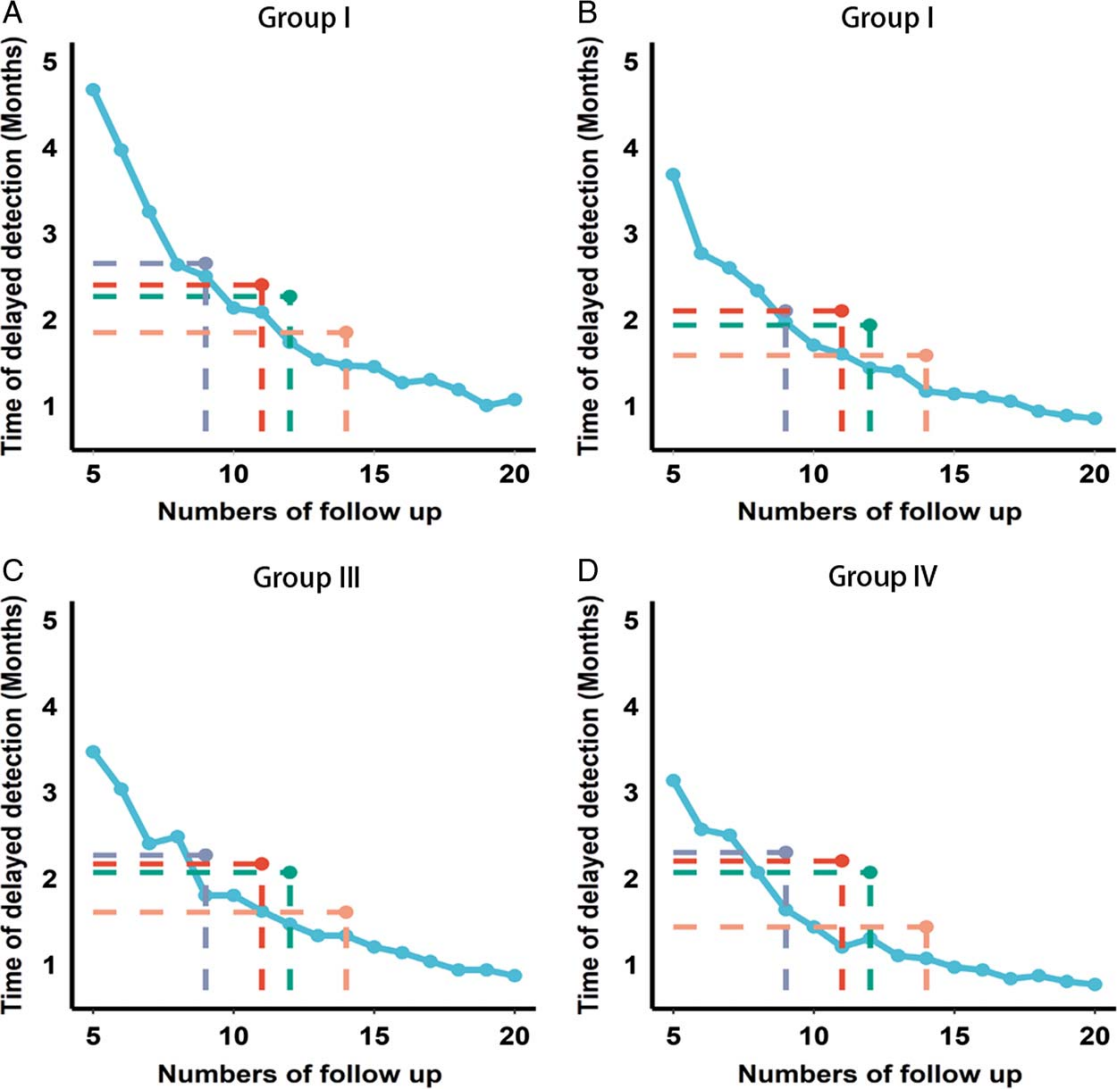
Comparison of performance between different strategies in each group. Average delays in detection of disease failure in risk-based surveillance arrangements (blue curve) and the control follow-up strategies (the gray, green, red, and pink dots, respectively, represent the follow-up protocol issued by CROSS, NEO5010, NCT1596, and NCCN) for patients in group I (A), group II (B), group III (C), and group IV (D).

Taking advantage of the RSF model^31^, we designed follow-up strategies based on cumulative recurrence probabilities. We assumed the visits in the first 5 years ranged from 5 (one visit per year on average) to 20 (one visit per 3 months on average), and arranged the visits for four groups according to the cumulative risk of recurrence. Then, we calculated the DDT of the follow-up strategies in each group to compare the surveillance performance of the RSF-based arrangements and other strategies recommended by guidelines or clinical trials. As expected, the median DDT sharply decreased when the number of visits increased, and the downward trend gradually became smoother as the number of visits exceeded 10 (Fig. 2). The gray, green, pink, and red dots represented the median DDT of the follow-up protocols issued by the guidelines and clinical trials^2,23–25^. As we observed, the quantitative RSF-based strategies outperformed previous experience-based strategies, mainly reflecting in the significantly reduced DDT with the same number of visits to the control strategies. In groups I, II, and III, the RSF-based arrangements consistently gained outstanding performances, with the DDT of 12 visits lower than those of 14 visits from the NCCN strategies (Fig. 2A–C). In group IV that represented more advanced diseases, the RSF-based strategy showed greater advantages that achieved comparable DDT to the NCCN strategy with just 10 visits across 5 years (Fig. 2D).

### Recommendation of RSF-based surveillance strategies

**Figure 3:**
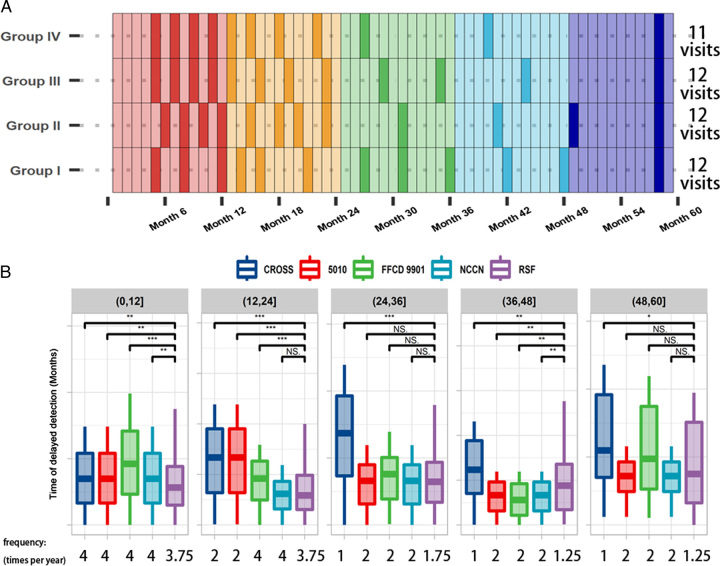
Recommended follow-up schedules for patients in each group and their surveillance capacities from year 1 to 5. (A) Follow-up schedules for EC patients in different groups based on RSF. The opaque color blocks represented a scheduled outpatient visits in that month. (B) Delayed detection time of the follow-up strategies across different time periods. Symbols: NS, *, **, and *** standed for *P*≥0.05, *P*<0.05, *P*<0.01, and *P*<0.001.

Next, we sought to find optimal follow-up arrangements. Referring to the most intensive NCCN strategies, we adopted the schedules that used fewer visits and gained lower DDT than the controls (Fig. 3A). During the first 5 years after surgery, we recommended 12 visits for both group I (3, 3, 3, 2, and 1 per year), II (4, 4, 1, 1, and 2 per year) and III (4, 4, 2, 1, and 1 per year). Compared to groups I–III, recurrence events in group IV were more concentrated within the first 2 years (Fig. 1B), therefore we suggested 11 visits for group IV (4, 4, 1, 1, and 1, respectively).

To further understand how our developed strategy worked, we calculated the DDT across five time points and used the Wilcoxon signed-rank test to compare the DDT of diverse strategies in different time periods (Fig. 3B). Using 11.75 visits on average per group, our recommended schedules showed noninferior DDT than the other four widely used strategies in the first 2 years, the time which covered around three-quarters of the events, and prominently outperformed their counterparts in the 1st year. Note that patients with recurrences within 2 years had worse postrecurrence survival than those undergoing late recurrences (Figure S3A). Although the DDT of our strategy was higher in the 4th year than the NCCN strategies, it was similar to that of the NCCN strategies (14 visits on average) in the 3th and 5th years. These results demonstrated that our risk-adaptive strategy reduced DDT mainly by timely detection of events around the recurrence peaks.

### Validation of the recommended follow-up schedules

First, we employed bootstrap validation to evaluate whether our proposed strategies worked uniformly well in different patient populations. In 100 repeats of bootstrap resampling, we calculated the DDT of each strategies in the simulated dataset and found that our strategies detected events more timely in all repeats (Figure S4A), averagely lowering the DDT by 5–25% in four groups when comparing with the NCCN guideline. Notably, the risk-based strategies yielded greater improvement in recurrence detection as the recurrence risk increased in four groups (Figure S4B).

Additionally, we assessed the performance of our recommended strategies in an independent cohort by applying different follow-up strategies and calculating the DDT. With a median follow-up time of 50.3 months (95% CI: 46.4–54.6 months), 210 recurrence events occurred in the validation cohort. Although the demographic and clinical characteristics of the validation cohort were dissimilar from those of the training cohort (Table S2), the risk-based surveillance strategy detected recurrence events more timely than other strategies did in the validation set in terms of DDT, indicating excellent applicability and superiority of the risk-based strategy (Table S4).

Theoretically, the ideal follow-up strategy also required a better cost-effectiveness, which meant detecting more events using fewer visits. We constructed the Markov decision-analytic model to evaluate the cost-effectiveness of different strategies and the model parameters were summarized in Table S5. The lower incremental cost-effectiveness ratio (ICER) represented that an intervention could improve patient survival at a lower cost. In group I, the ICER for the 5010, the NCCN, and the NCT03001596 surveillance strategies were $10724/QALY (quality-adjusted life year), $13442/QALY and $13187/QALY, respectively, the risk-based strategy was $8896/QALY while setting the CROSS strategy as a reference while setting the CROSS strategy as a reference (Table 1). For the remaining patient groups, the risk-based strategy remained the most cost-effective, with ICER gradually decreasing from group II to IV ($7945/QALY, $5738/QALY, and $3926/ QALY, respectively). These results indicated that our recommended strategy was more economical and exerted higher efficiency in patients with advanced diseases.

**Table 1:** Cost-effectiveness analysis using Markov models.

	Cost ($)	Incremental cost ($)	Effectiveness (QALY)	Incremental effectiveness	ICER ($ per QALY)
Group I					
CROSS	5772	Reference	42.010	Reference	Reference
NEOCRT5010	6898	1126	42.115	0.105	10724
NCCN	7869	2097	42.166	0.156	13442
NCT03001596	7882	2110	42.170	0.160	13187
Risk-based strategy	6964	1192	42.144	0.134	8896
Group II					
CROSS	7029	Reference	38.785	Reference	Reference
NEOCRT5010	7976	947	38.887	0.102	9284
NCCN	8724	1695	38.969	0.184	9212
NCT03001596	8820	1791	38.983	0.198	9045
Risk-based strategy	8181	1152	38.930	0.145	7945
Group III					
CROSS	8680	Reference	34.535	Reference	Reference
NEOCRT5010	9486	806	34.665	0.130	6200
NCCN	10175	1495	34.769	0.234	6389
NCT03001596	10355	1675	34.798	0.263	6369
Risk-based strategy	9839	1159	34.737	0.202	5738
Group IV					
CROSS	9984	Reference	30.578	Reference	Reference
NEOCRT5010	10688	704	30.727	0.149	4725
NCCN	11361	1377	30.879	0.301	4575
NCT03001596	11598	1614	30.919	0.341	4733
Risk-based strategy	10891	907	30.809	0.231	3926

ICER, incremental cost-effectiveness ratio; QALY, quality-adjusted life years.

### Recurrence patterns of EC and implications for surveillance

**Figure 4:**
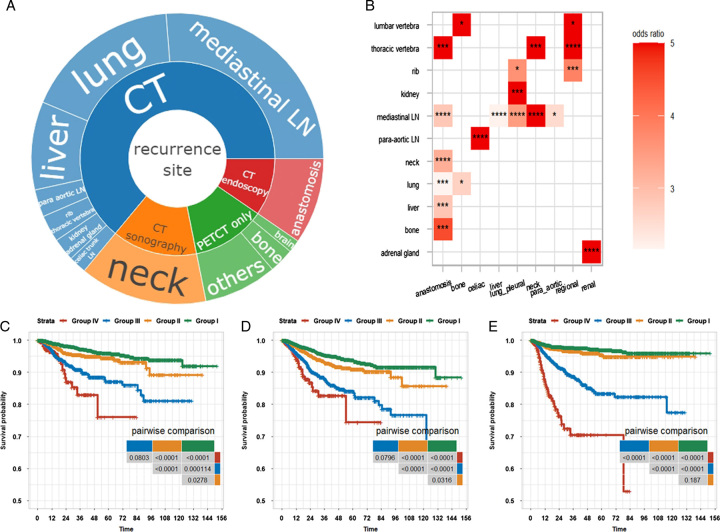
Recurrence pattern of EC patients and cumulative risks of specific events of patients in different groups. (A) Distribution of recurrence sites and corresponding detection methods. (B) Co-occurrences of site-specific recurrence events. Fisher’s exact test was used to measure whether metastasis from two organs occurred in a patients by chance or not. Symbol: ʻ*ʼ: FDR ≤0.05, ʻ**ʼ: FDR ≤0.01, ʻ***ʼ: FDR ≤0.001. (C–E) Site-specific recurrence free survival curves of the four different groups, stratified by anatomatic locations, such as anastomosis stroma (C, endoscopy), neck (D, CT of neck and ultrasonography), and systemic metastasis only detected by PETCT (E).

Taking advantage of our large real-world EC database, we tried to depict the organ-specific patterns of EC recurrences. In line with previous reports^10^, the lung (30.8%), liver (17.8%), and neck (25.3%) were the three most common locations of DM. 86.2% of patients had recurrence events that occurred in organs covered by CT of the neck, chest, and upper abdomen, while about 17% of events were firstly detected by PETCT (Fig. 4A). We did not observe remarkable temporal distinctions of recurrences in different sites (Figure S3B/C). We further asked whether the emergence of metastasis co-occurred by chance or not. After multiple test corrections of the result from the Fisher’s exact test (FDR<0.05), we found some co-occurrences from proximal anatomic locations, such as the kidney and adrenal gland (Fig. 4B). Furthermore, the LRR in the chest often occurred simultaneously with the neck and liver metastasis. Our results suggested that examination tools on the neck were also needed to comprehensively evaluate the recurrences of EC patients.

Moreover, we analyzed the site-specific DFS to investigate the utility of different surveillance methods in different groups. As shown in Figure 4 C–E, the recurrence risk of anastomosis stroma and neck metastasis were higher in patients in groups III and IV than those in groups I and II. As for hematogenous metastasis outside anatomic sites covered by the CT of the neck, chest, and upper abdomen, we found that these events were rarely seen in groups I and II, but more common in patients with more advanced diseases (Fig. 4E). Therefore, monitoring of DM by a more aggressive method such as PETCT is an option in the first 3 years for patients in group IV, while for patients in group III, the monitoring should be continuous in year 1–5 after treatment.

## Discussion

Given the high recurrence potential that nearly a half of EC patients will eventually die of this disease^3–5^, active and customized surveillance is essential for timely salvage treatment upon recurrences to improve patient survival and quality of life^1^. A number of guidelines suggested identical follow-up schedules for all patients, which may cause over-utilization or under-utilization of testing for certain patient subsets^32,33^. There was no data to evaluate the effectiveness of these guidelines. In this study, we designed follow-up schedules for different risk stratifications. Through validations, our proposed strategies were more efficient and economical than the NCCN guidelines and several follow-up protocols from clinical trials.

### Development of recurrence risk stratification

As the recurrence risk of EC varied across populations with different pathological features, we integrated four widely adopted variables for patient stratification: LVI, PNI, pathological N stage, and LNR. Similar to previous findings, LVI or PNI were critical determinants of tumor recurrences in $T_{\mathrm{any}}N_{0}M_{0}$ populations^13,34,35^. Another variable, LNR, subdivided $N_{1}$ patients and the recursive partition algorithm suggested 0.05 as the optimal cutoff, which was similar to a previous study^13^. These results indicated that the N stage of these $N_{1}$ patients was underestimated, because they had lower LNs examined compared to patients with LNR ≤0.05^17,36^ (Figure S2B).

### Customized follow-up strategies for different patient groups

Theoretically, the surveillance might be as frequent as possible for EC patients, as salvage treatment on localized recurrence and oligometastasis can achieve more favorable outcomes^6,37^. Obviously, this ideal situation is insufferable, because of the relatively poor economic condition of EC patients^38^. Therefore, the design of follow-up schedules requires a better compromise between expenses and efficiencies. Based on the recurrence risk stratification, we estimated the recurrence probability for each groups and assigned the outpatient visit to the time when relapse events probably occurred. Timely detection of recurrences owing to our follow-up schedules might also facilitate a more accurate estimate of DFS, which might have great implications on those clinical trials focusing on DFS^8^. Performance evaluations demonstrated that our proposed strategy was more efficient than the guideline strategies, especially at the peaks of recurrence, and more economic that may contribute to the reasonable conservation of medical resources.

Another study reported that 45 and 60% of the hospitals in Japan recommended less than 2 visits with medical imaging in the first and second years after surgery^32^. Our results indicated that, during the recurrence peaks (within 2 years), four visits per year were needed to detect disease progression in time and launch salvage therapies^30^. Our data also suggested elongation of the course of adjuvant therapy from 1 years to 2 years in order to cover the whole recurrence peaks^39^.

### Implications of surveillance tools for different groups

As opposed to the NCCN guidelines, which recommended the CT of the chest and abdomen as regular detection tools^2^, our data indicated that CE-CT screening from the neck to the upper abdomen should be a fundamental tool for oncologic surveillance because up to 80% of events could be detected by CT. Other than supraclavicular LN, lung, and liver metastasis, our data demonstrated that about 20% of events initially occurred at anatomic sites not covered by CT from the neck to the upper abdomen. Henceforth, PETCT might be a viable choice for these patients, especially for patients in groups III and IV, though it was not recommended as a regular weapon in NCCN guidelines^2^. Though metastatic EC was usually uncurable, early detection, and treatment of oligometastasis could improve survival, with the 3-year survival rate of ~30% in patients with isolated metastasis, while the survival rate is only 10% in patients with multiple lesions^7,37^.

Our study was limited because the data was collected retrospectively. The widely used follow-up protocol in clinical practice recommended identical follow-up intervals, which resembled the current guidelines. Asymptomatic tiny recurrences could be detected near the scheduled time, which might overestimate the performance of the control strategies. Nevertheless, our recommended strategies were generally more efficient and the multicenter prospective cohort was needed to validate our results. Secondly, outpatient visits for other purposes including screen for adverse complications like anastomotic stenosis were not considered in our study because recurrence was the top concern for EC patients. Thirdly, the molecular classifications of EC have been proven to be reliable predictor of prognosis and should be considered in future researches^40^.

## Conclusion

In summary, we developed and validated recurrence risk stratifications for EC patients with radical surgery. We further devised follow-up schedules that achieved more timely detection of recurrences with fewer visits. The recommended surveillance schedules reduced the number of follow-up visits by 17% and were still more effective and economical than the NCCN surveillance strategies.

## Ethical approval

The study protocol was approved by the Ethics Committee of SYSUCC (B-2023-428).

## Sources of funding

This research was funded by the National Natural Science Foundation of China (No. 82272881/82273032/82072607).

## Author contribution

J.F., Q.L., and H.Y.: conceptualization; Z.M. and J.X.: methodology; Z.M., X.X., J.W., and C.L.: data curation; J.X. and Z.M.: formal analysis; Z.M., J.X., and C.L.: writing original draft; J.F. and Q.L.: writing – review and editing; Z.M. and H.Y.: manuscript revision; Z.M. and J.X.: visualization; J.F.: supervision; J.F., J.W., and H.Y.: funding acquisition. All authors have read and agreed to the published version of the manuscript.

## Conflicts of interest disclosure

There is no conflicts of interest to state.

## Research registration unique identifying number (UIN)

1. Name of the registry: Research registry.
2. Unique identifying number or registration ID: researchregistry 9519.
3. Hyperlink to your specific registration (must be publicly accessible and will be checked): researchregistry.com/browsethe/registry#home/registrationdetails/65027500dc8eae00288744d2/.

## Data availability statement

The datasets involving patients’ clinical characteristics can be available upon reasonable request from the corresponding author. The study’s authenticity has been validated by uploading the key raw data onto the research data deposit (RDD) public platform (http://www.researchdata.org.cn).

## Guarantor

Zihang Mai, Jianhua Fu.
